# Synthesis and Study of Morphology and Biocompatibility of Xanthan Gum/Titanium Dioxide-Based Polyurethane Elastomers

**DOI:** 10.3390/polym13193416

**Published:** 2021-10-05

**Authors:** Shazia Naheed, Muhammad Shahid, Rashida Zahoor, Zumaira Siddique, Nasir Rasool, Sajjad Haider, Shaukat Khan

**Affiliations:** 1 Department of Chemistry, Government College University, Faisalabad 38030, Pakistan; alchemist019021@gmail.com (M.S.); rashidazahoor786@gmail.com (R.Z.); zumairasiddique@gmail.com (Z.S.); nasirrasool@gcuf.edu.pk (N.R.); 2 Chemical Engineering Department, College of Engineering, King Saud University, P.O. Box 800, Riyadh 11421, Saudi Arabia; 3 School of Chemical Engineering, Yeungnam University, 280-Daehak-Ro, Gyeongsan 712-749, Korea; shaukat85@yu.ac.kr

**Keywords:** polyurethanes, xanthan gum, titanium dioxide, atomic force microscopy, X-Ray diffraction

## Abstract

A series of xanthan gum/titanium dioxide-based polyurethane elastomers were synthesized through the prepolymer method by the step growth polymerization. In the present work, xanthan gum was used as a bioactive material, with TiO_2_ as a nanofiller. The structural characterization of newly prepared polyurethane samples was carried out with the help of Fourier Transform Infrared Spectroscopy. Thermogravimetric Analysis gave us the information about the thermal stability. Differential Scanning Calorimetry directs the thermal changes in the polyurethane samples. The Atomic Force Microscopy technique revealed that the degree of micro-phase separation increases by augmenting the % age of TiO_2_, which was further confirmed by X-Ray Diffraction results. XRD confirmed the crystallinity of the final sample at about 2θ = 20°. Antimicrobial activity determined through the Disc Diffusion Method, and the results indicated that the synthesized polyurethane have antimicrobial activity. The water absorption capability of the polyurethane samples showed that these polymer samples are hydrophilic in nature.

## 1. Introduction

Polyurethanes have become the 6th most used group of polymers in the last few decades and have gained more importance due to multiple use in different fields, such as coatings, adhesives, furniture, and foams [1,2]. Polymeric composites are currently at an important crossroad, where research is shifted toward more sustainable bio-based materials. In the last few years, biomaterials and biocomposites have gained extraordinary attention. Biocomposites have additional benefits, apart from their eco-friendly nature [3]. Polyurethanes are often applied as biomaterials because they have good mechanical properties and low adsorption of biomolecules [4].

In recent years, numerous polymeric materials have utilized for the manufacturing of different medical devices that interact with blood, body liquids, and tissues [5]. The soft, as well as hard, segment of polyurethanes exhibited elastomeric properties [6]. Basically, the polyurethanes are incorporated in medical applications due to their toughness, cost effectiveness, and durability [7,8,9].

By comparing them with additives, they have gained much more attention due to ease of processing and, specifically, the countless architectural variety for optimizing their stages. The characteristics of the final polyurethane could be modified and controlled by the addition of additives [10]. They have a basic skeleton for lightweight materials, which is necessary for the transport industry. Reducing average weight decreases fuel usage, as well as greenhouse gases. Moreover, most of them are recyclable, bioactive, and biodegradable, depending upon the ingredients [11]. The physical properties of a polyurethane, such as density, tensile strength, high water abrasion, etc., distinguish its functioning [12]. They have admirable properties, such as hardness, tensile, compressive, impact resistance, etc. In general, polyurethanes are used in many different applications. These properties are tightly correlated with the biphasic nature of the segmented polyurethanes in the hard and soft phase. This, in turn, depends upon the chemical nature and composition of both phases. Flexible segment polyether and polyester-based polyurethanes (PUs) are susceptible to degradation under hydrolytic and oxidation environments [13].

The mechanical characterization of microcapsules of polyurethanes exhibits increased mechanical strength by a variable quantity of chain extenders, i.e., 1,4-butanediol (BDO) [14]. In the modern era, the standard of living could be enhanced with the employment of polymeric biomaterials. Generally, such biomaterials are simulated/artificial polymers, which are used in artificial organs, implants, dentistry, abrasion bandages, and drug delivery systems [15]. However, investigation on the degradation, morphology, and thermal and mechanical behavior is crucial to determine the end use of these materials.

In various extra and intracorporeal devices, biomedical polyurethanes are extensively applied. Typical illustrations include pacemaker leads insulation, indwelling tubes, heart pump tubes, and balloons of angioplasty [16]. Aliphatic diisocyanates, such as hexamethylene diisocyante (HDI), or cycloaliphatic diisocyanates, such as 4,4′-methylenebis(cyclohexyl isocyanate) (H_12_MDI) and isophorone diisocyanate (IPDI), have bid superior stability over the aromatic isocyanate. The aliphatic diisocyanates show improved phase separation compared to corresponding aromatic diisocyanates. They also show improved phase separation behavior over the corresponding aromatic diisocyanates [3]. Today, extensive studies have been accomplished on polyurethane due to their inimitable possessions, as well as to illustrate their different behaviors [17].

By supplementing the diols of lower molecular weight as chain extender, Barikani and Hepburn reported that thermal stability of polyurethanes was enhanced [18]. Polyurethane biocompatible organic polymers, with an alginate nucleus and chitosan shell, used as nanoparticles, were prepared for increasing encapsulation efficiency, more regular insulin liberation, and better insulin accessibility [19]. Today, polysaccharides extracted from plants, such as guar gum or pectin, and extracted from algae, such as alginate, are replaced by some bacterial exopolysaccharides, such as xanthan gum and gellan gum [20]. *Xanthomonas campestris* produces the xanthan gum, which is basically heteropolysaccharide. Xanthan gum has extensive applications in industries, such as food, oil, pharmaceuticals, etc., due to its rheological properties, i.e., pseudo plasticity, high viscosity, etc. [1].

Biopolymer collection has been turned into a noteworthy natural issue today because of expanding use of biomaterials for pharmaceutical applications. Reprocessing of polymeric wastes by recycling them has been extensively applied. Now, there is a dire need to synthesize environmentally friendly biodegradable polymer [15].

Biocompatibility is an index which is a basic requirement for a polymeric material to be appropriate as an ideal biomaterial, which, in turn, depends upon degradable nature, cytotoxicity, and the other mechanical properties [21].

A few biomaterials out of bulk commodity polymers have been found promising. The novel polymeric materials of nanocomposites of nylon 6/clay has led to emanation having unanimity of incomparable characteristics [22]. For structural development, the amalgamation of layered nanofillers can radically influence the blends’ and polymers’ microphase morphology by acting as templates [23]. In order to promote biodegradability and biocompatibility, current studies have marked the porosity worthy of acritical stuff, more distinctively, porosities distribution and structure [24].

In this study, the xanthan gum/TiO_2_-based polyurethanes (XTPUs) were prepared by augmentation of weight (% age) of TiO_2_, by the prepolymer method. The microstructure biocompatibility of the nanocomposites was investigated. It was hoped that the introduction of nano TiO_2_ could not only improve the physical properties and biocompatibility of PU but also inhibit the growth of bacteria even when nanofiller TiO_2_ were used in low concentrations and were embedded in the polyurethane (PU) matrix.

## 2. Experimental

### 2.1. Materials and Synthesis

Analytical grade chemicals were used in this research work, provided by Sigma Chemical Co. (St. Louis, MO, USA), including Hydroxyl-terminated polybutadiene (HTPB, Mn = 3000 g/mol); Isophorone Diisocyanate (IPDI), 1,4-butanediol (BDO) was used as chain extender, titanium dioxide (TiO_2_) was used as a nanofiller, and the commercial xanthan gum (XG) was used as bioactive material. These were used to synthesize polyurethanes’ elastomers by the step growth synthesis method. Before the use of these chemicals, for removal of moisture, they were dried at 80 °C in an electrical oven. During the drying process, they were placed in a vacuum for 24 h.

The prepolymer synthesis was done, according to a reported method [25]. Firstly, TiO_2_ (% by weight) and XG (% by weight) were dispersed and mechanically stirred in one mole of HTPB for 2 h at 100 °C until it completely dispersed in four-necked specially-designed apparatus equipped with round bottom flask, heating oil bath, magnetic stirrer, reflux condenser, nitrogen inlet, and dropping funnel at 100 °C. Then, it was reacted with two moles of IPDI for 2 h at 80–100 °C, in order to obtain isocyanate terminated (NCO) polyurethane prepolymer. The stirring continued until NCO-terminated prepolymer was synthesized. Titration with n-butylamine (ASTM D 2572-80) was conducted to obtain the NCO contents of the polymer. The synthesis of prepolymer was confirmed using FT-IR spectroscopy [26].

### 2.2. Synthesis of Final Polymer

**Figure 1:**
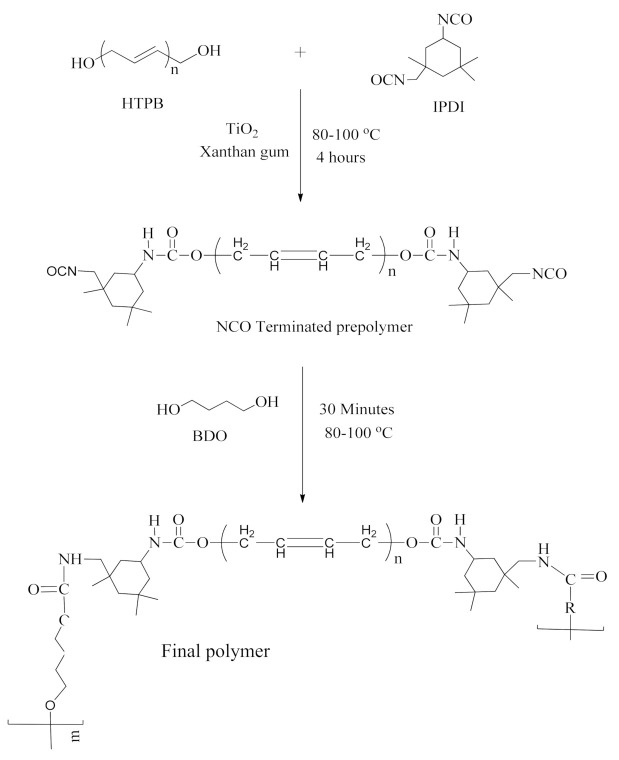
Synthesis of polyurethane.

The final xanthan gum/TiO_2_-based polyurethane (XTPU) polymer was obtained by stirring, for 30 min, the polyurethanes prepolymer with the chain extender 1,4-butanediol (1.2 mol). On the appearance of homogeneity and completion of dispersion of the chain extender in the reaction mixture, the polymer, in liquid form, was poured on the Teflon plate in order to develop a sheet having thickness of almost 2–3 mm. Then, at 100 °C for 24–48 h, the circulating hot air oven was used to cure the synthesized polymer. Before testing, i.e., characterization by various techniques, the cured sheets was stored at 25 °C for one week in order to attain almost 40% humidity. A series of six polymer samples were prepared by keeping constant the weight % age of XG, i.e., 1% and by varying weight % age of TiO_2_ from 0% to 5%, as shown in Table 1. Figure 1 presents the schematic demonstration of the chemical route for synthesis of the polymer.

## 3. Characterization

### 3.1. Fourier Transform Infrared Spectroscopy (FT-IR)

Structural characterization of synthesized polymer was done by BRUKER TENOSR II FT-IR spectrometer (Bruker, Billerica, MA, USA). The infrared spectra recorded after 15-s interludes, by using 8 scans over a resolutions of 2 cm^−1^. The KBr beam splitter and DTGS detector were provided to the spectrometer. Data was collected, processed, and presented by thermo scientific spectroscopy software against a time-dependent series. The mechanism of reaction, as well as crosslinking behavior, was interpreted by FT-IR [27].

### 3.2. Atomic Force Microscopy (AFM)

AFM (CP-II, Veeco, Newport Beach, CA, USA) was used to assess surface morphology of prepared samples. A phosphorus-doped silicon-integrated pyramidal tip was used as support, in order to acquire images in tapping mode with a triangular cantilever (force constant of 20–80 N/m). Simultaneously, the images of topography and phase separation were recorded. The root-mean square average of the surface roughness was calculated within the given area as the standard deviation of all heights. Image Pro Plus 4.5 software (Media Cybernetics, Rockville, MD, USA) was used to measure the average hard and soft domain size from the phase images [28].

### 3.3. Thermogravimetric (TGA) and Differential Scanning Calorimeter (DSC)

Thermogravimetric and Differential Scanning Calorimeter analysis were accomplished using an SDT Q600 V20.9 Build 20 (TA Instruments, Newcastle, DE, USA) under a dry nitrogen flow. The temperature range was set at 0–600 °C, and tamp (heating) rate was retained at 10 °C/min. The samples for TGA and DSC were primed on a glass substrate by spin coating of the mixed solution. Then, it was cured at various temperature steps. Aluminium sample pans were utilized to seal the almost 4 mg of each sample. Then, the prepared materials were analyzed under dry nitrogen by DSC over a heating rate of 10 °C/min from 0 to 500 °C. The glass transition temperature (Tg) crystallization temperature (Tc) and melting temperature (Tm) of the prepared polymers were investigated with the help of differential scanning calorimeter SDT Q600 V20.9 Build 20 [29].

### 3.4. Antimicrobial Activity

The capacity of a substance to have contact with the human body tissues without affecting the human body is called biocompatibility. The samples were subjected to assess the antimicrobial activity. The inhibition studies were done by actively growing bacterial cells. First of all, a nutrient agar media of 1000 mL was prepared, and 150 mL was poured in separate 150-mL flasks. The autoclave was used for 15 min at 120 °C by putting in agar medium nutrients flasks. Later on, it was cooled and bacteria, i.e., *Escherichia coli* (gram-negative) and *Macrococcus* (Gram-positive), were added to about 15 µL in the above two flasks. The sterile petri plates were used, and almost 20 mL of each agar nutrient medium was transferred in these plates, while room temperature was maintained. For 24 h, the samples were incubated at 37 °C, and they were then placed in petri dishes. The zone of inhibition, in which growth of bacteria was inhibited, was calculated by the diffusion active compound in the surrounding of the sample [30,31].

### 3.5. X-ray Diffractometry (XRD)

In X-ray diffractometry, X-rays were used to irradiate XTPU polymers, and the scattering pattern was noted. Basically, the scattered radiations intensity noted as a function of the scattering angle θ. The X-rays scattering is due to electron density difference. A small angle scattering of X-rays is used to investigate the microstructures in the range of tens to thousands angstrom (Ǻ). It is basically a phase segregation in polymers. Meanwhile, X-ray scattering of a wide angle was also applied to calculate the crystallinity of the polymer samples at atomic level. The different reflections of XRD were the result of crystalline behavior of the polymer samples, which was calculated by using Bragg’s law.

2*d* sin θ = *n*λ, (1)

where *d* is the basically distance between crystalline planes, θ is the angle of X-ray beam which it makes with the planes, *n* is an integer, and λ is the wavelength. The dispersion range (2θ) of 0–70° was used to record the relative intensity [32].

## 4. Results and Discussion

### 4.1. FT-IR Analysis

**Figure 2:**
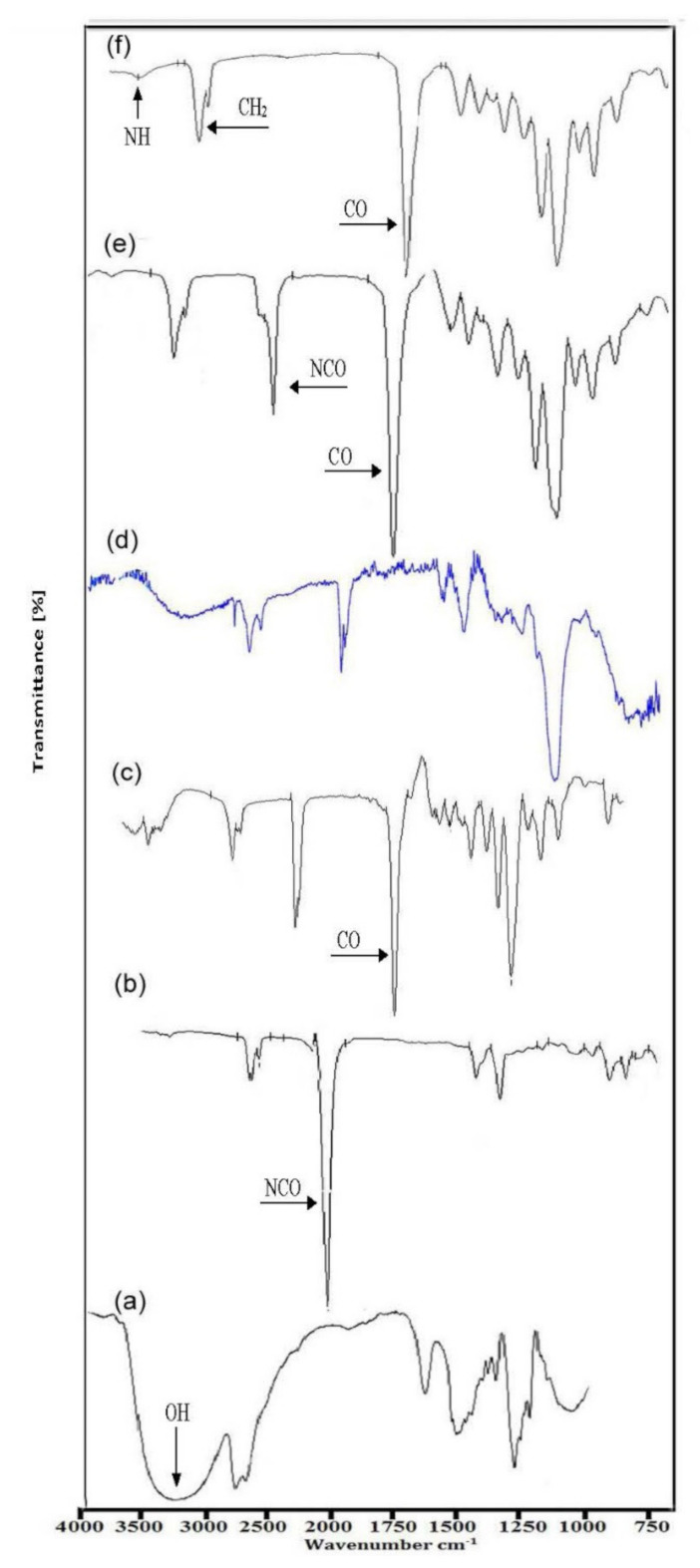
FT-IR spectra of (**a**) 1,4-Butanediol (BDO), (**b**) Isophrone diisocyanate, (**c**) Hydroxyl Terminated Polybutadiene (HTPB), (**d**) Xanthan Gum (XG), (**e**) NCO-terminated Prepolymer, and (**f**) XTPU final Polymer.

Figure 2 represents the FT-IR spectra of 1,4-butanediol, Isophrone Diisocyanate, HTPB, Xanthan Gum, NCO-terminated Prepolymer, and XTPU final Polymer. Figure 2a shows the FT-IR spectrum of 1, 4-butanediol and indicated that, due to the OH group, a very strong peak appeared at 3400 cm^−1^. Due to the CH_2_ group the peak appeared at 2900 cm^−1^. In Figure 2b, the FT-IR spectrum of IPDI showed the anti-symmetric stretching peak of the CH_2_ group at 2939.52 cm^−1^ and symmetric stretching peak of the CH_2_ group at 2862.0 cm^−1^ was also observed. In the spectrum of IPDI, due to the NCO group, a sharp peak at 2250.71 cm^−1^ was also observed. In Figure 2c, the FT-IR spectrum of HTPB where the peak of hydroxyl (OH) appeared at 3736.12 cm^−1^ due to stretching vibration is shown. There appeared a peak due to anti-symmetric stretching vibration of the CH_2_ group, observed at 2945.30 cm^−1^. Figure 2d shows the most significant bands for the xanthan gum in the range of 4000–500 cm^−1^. It includes an axial deformation of OH at 3300–3450 cm^−1^ and an axial deformation of C-H at 2855–2926 cm^−1^, which may due to absorption of symmetrical and asymmetrical stretching of -CH_3_ or may be due to -CH_2_-groups. There was also an aldehydic (-CHO) peak at 1710–1730 cm^−1^. An axial deformation of C-O of enols was observed at 1530–1650 cm^−1^. An axial deformation of C-O at 1045–1150 cm^−1^ was also observed. In Figure 2e, the NCO-terminated prepolymer clearly showed that peaks due to the OH group diminished. The peak due to the NH group appeared at 3325 cm^−1^. The NCO group’s intensity was lower to some extent, which means that isocyanate groups reacted completely. The formation of the prepolymer was confirmed by the appearance of peak of the NH group at 3325 cm^−1^ and supported its proposed structure. The anti-symmetric peak of the CH_2_ group was seen at 2945.30 cm^−1^. The stretching peak of –C=O was observed at 1724.36 cm^−1^. Figure 2f shows, by extending prepolymer with 1,4-BDO, that the FT-IR spectra showed a very strong peak at about 1707 cm^−1^, which was assigned to C=O stretching of urethane. Peaks corresponding to the absorption of NH, C=O, and C=O were observed at 3325 cm^−1^, 1707 cm^−1^ (non-hydrogen bonded), 1643 cm^−1^ (hydrogen bonded), and 1225 cm^−1^, respectively, which indicate the new synthesized product being in the urethane group. The observed N-H bending vibrations at 1598 cm^−1^, C-O-C stretching absorption band corresponding to linkage between OH and NCO groups to form urethane bond in the range 1057–1130 cm^−1^, also provide strong evidence for the formation of XTPU.

**Figure 3:**
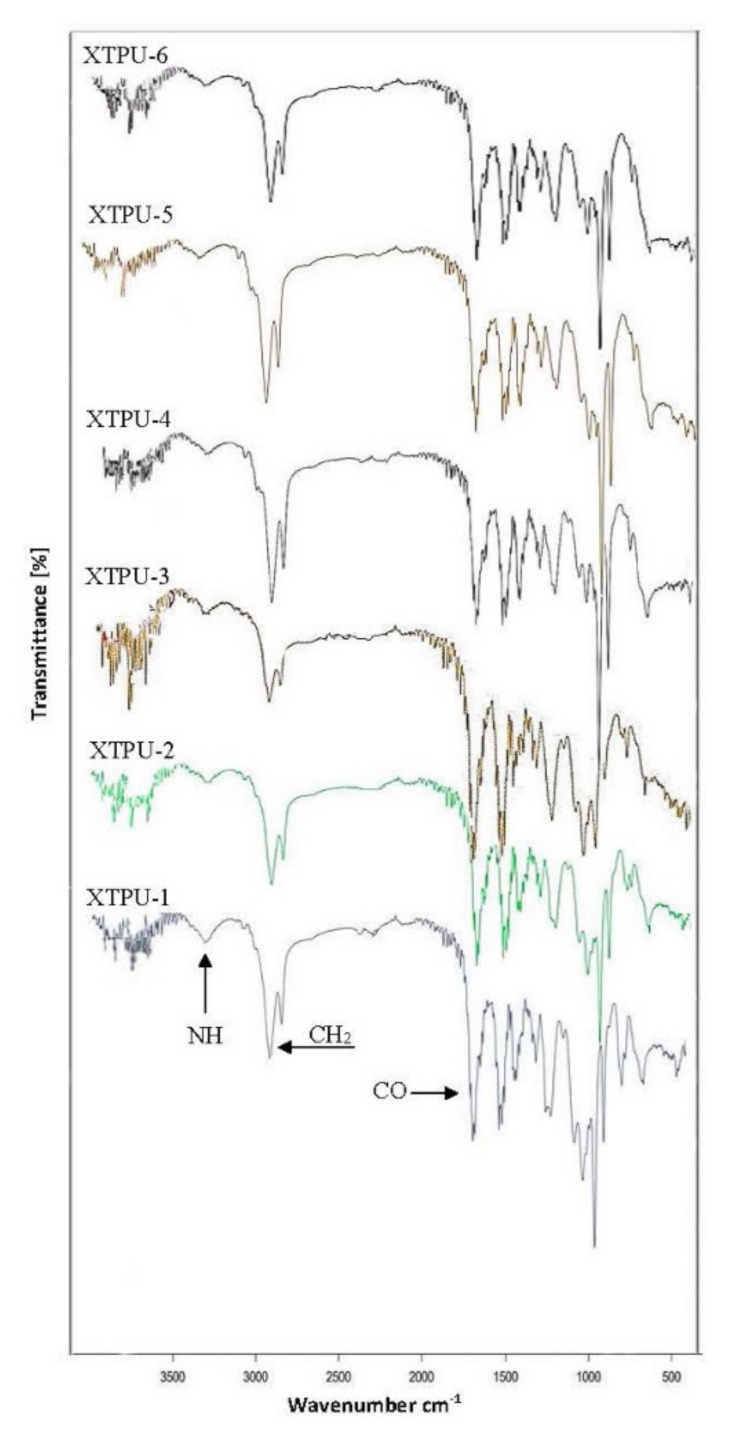
FT-IR spectrum of polyurethane samples XTPUs having 0–5% TiO_2_, respectively.

The FT-IR spectra of XTPU 1 to 6 with varying weight % age of TiO_2_ and constant weight % age, i.e., 1% xanthan gum, are shown in Figure 3. All the spectra confirm the formation of urethane linkage in the final XTPU polymer samples. In Figure 3, the XTPU-1 spectra showed the formation of urethane linkage NH at the peak 3750.17 cm^−1^, by the disappearing peak of NCO at 2156 cm^−1^. It showed the symmetric and asymmetric peak at 2840 cm^−1^ of the –CH_2_ group and 2913.45 cm^−1^, respectively. The peak of –C=O group appeared at 1697.82 cm^−1^. FT-IR spectra of XTPU-2 is shown in Figure 3. This spectra showed the formation of urethane linkage NH at the peak 3750 cm^−1^. It also displayed the symmetric and a-symmetric peak of the –CH_2_ group at 2800 cm^−1^ and 2912.59 cm^−1^. The –C=O group peak appeared at 1698.02 cm^−1^. The peak of NCO disappeared at 2160 cm^−1^. The FT-IR spectra of XTPU-3 is shown in Figure 3. This spectrum characterizes the peak of –C=O group at 1698.02 cm^−1^. The peak of N-H forms at 3750.13 cm^−1^. It also gives the symmetric and a-symmetric peak at 2850 cm^−1^ and 2911.38 cm^−1^. Figure 3 shows the FT-IR spectra of XTPU-4. This spectrum characterizes the peak of –C=O group at 1698.02 cm^−1^. The peak of N-H forms at 3853.72 cm^−1^. It also gives the symmetric and a-symmetric peak at 2843.05 cm^−1^ and 2913.05 cm^−1^. In Figure 3, the FT-IR spectra of XTPU-5 shows the formation of N-H at 3760 cm^−1^. It shows the formation of –C=O peak at 1698.05 cm^−1^. It also shows the a-symmetric and symmetric peaks of CH_2_ group at 2950 cm^−1^ and 2840 cm^−1^. Furthermore, it gives information about the disappearing peak of the NCO group at 2300 cm^−1^. Figure 3 also shows FT-IR spectra of XTPU-6 and confirmed the formation of N-H at 3753 cm^−1^. Moreover, it illustrates the formation of –C=O peak at 1697.97 cm^−1^. It shows the a-symmetric and symmetric peaks of –CH_2_– group at 2912.56 cm^−1^ and 2843 cm^−1^. It also gives information about the disappearing peak of the NCO group at 2300 cm^−1^.

### 4.2. Evaluation of Antimicrobial Activity

The gram positive, as well as gram negative, bacteria and fungi were used for antimicrobial activity, so they can be classified as evaluation of antibacterial activity and evaluation of antifungal activity.

#### 4.2.1. Evaluation of Antibacterial Activity

**Figure 4:**
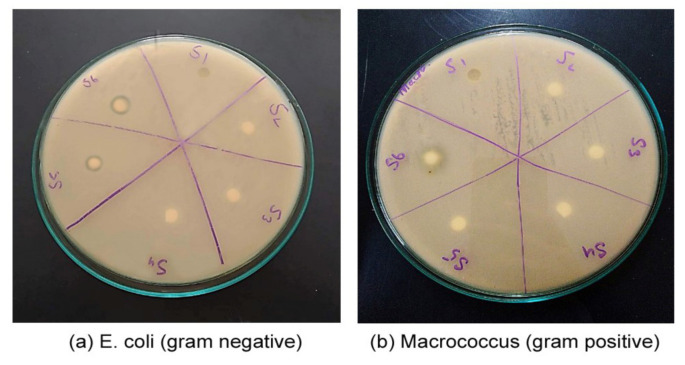
Antibacterial assay of samples against *E. coli* and *Macrococcus*.

Biocompatibility is necessary for any material to be in contact with living tissues without resulting any harm to living body. Most polyurethane polymers are biocompatible, and their biocompatibility was evaluated by antibacterial activity. Antibacterial analysis was carried out through the disc diffusion technique, and *Escherichia coli* and *Macrococcus* (a gram negative and gram positive, respectively) strains of bacteria were used for this purpose. On agar plates, the bacterial cultures were spread, and punched samples of polyurethane polymer of 5 mm diameter samples were applied over the plates. This complete setup was carried out at 37 °C in an incubator overnight. The results for biocompatibility with *E. coli* and *Macrococcus* are presented in Figure 4. The resistance of polyurethane samples against *E. coli* the gram negative and *Macrococcus* the gram positive bacteria are shown in the inhibition zone. It was found that antibacterial ability or biodegradability of a polymer depends upon the concentration of TiO_2_, along with content of xanthan gum used. We may increase the quality by changing the composition. It was observed that samples based on BDO show less antibacterial activity against *E. coli*. Antibacterial activity was influenced by bacterial strain used as given in results as reported in literature [30].

#### 4.2.2. Evaluation of Antifungal Activity

**Figure 5:**
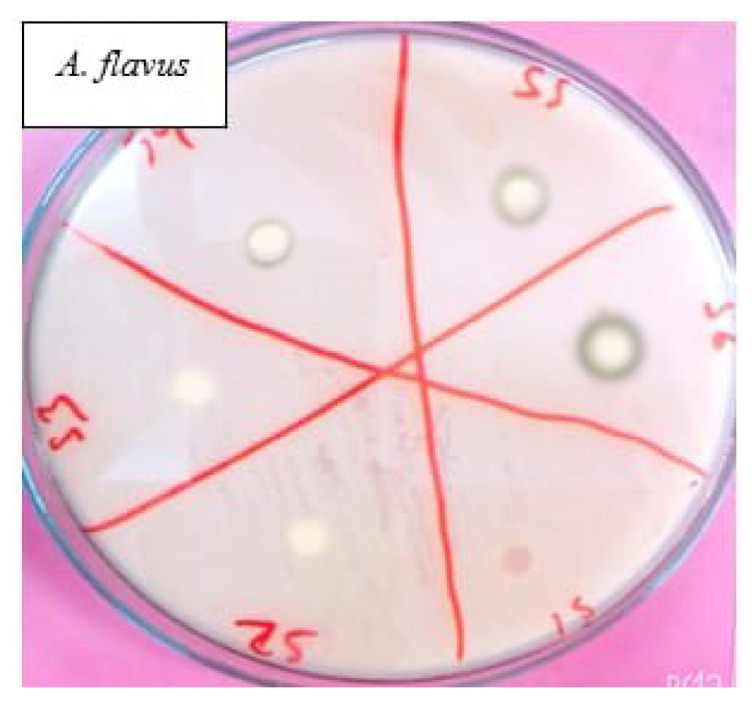
Antifungal assay of polyurethane samples against *Aspergillus flavus*.

Antifungal activity was evaluated against *Aspergillus flavus* using disc diffusion method. Vogel’s media was used for fungal culture growth. The selected polyurethane polymer samples were punched in disc of 5 mm diameter and applied over the place incubated at 37 °C overnight in incubator. Antifungal activity of 6 polyurethane samples with a variable content of Titanium dioxide and 1% xanthan gum was evaluated. The observed antifungal activity is shown in Figure 5. A clear zone of inhibition was observed on the plates of XTPU-5 and XTP-6; however, other polymer samples inhibited fungal growth at the area under the sample disc. The results exhibited that antifungal or biodegradability of polymer depends upon the content of TiO_2_ used. We may increase the quality by changing the composition. Moreover, the zone of inhibition can be increased by increasing the weight % age of TiO_2_.

### 4.3. Thermal Analysis

The thermal analyses of xanthan gum, titanium dioxide-based polyurethanes were performed by Thermogravimetric analysis (TGA) and Differential Scanning Calorimetry (DSC). These analyses are further explained below.

#### 4.3.1. Thermogravimetric Analysis (TGA)

**Figure 6:**
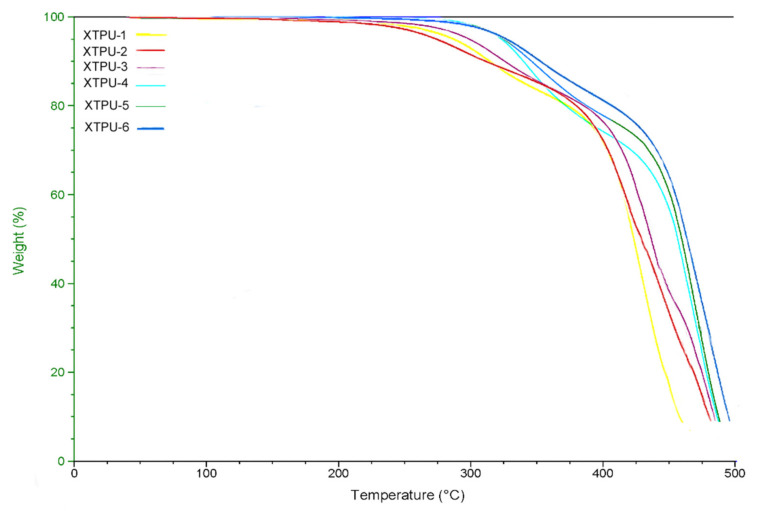
TGA thermogram of XTPUs at 500 °C.

The thermal properties of xanthan gum, titanium dioxide-based polyurethanes (XTPU) were analyzed by using the TGA technique. The TGA thermograms of XTPU 1 to 6 are shown in Figure 6. The thermograms of TGA of all XTPU samples showed thermal stability and thermal degradation behavior. In an inert atmosphere, the thermal analysis data revealed that all of the XTPU polymer samples are thermally stable up to 140–225 °C. All the polymer samples faced a 10% loss of weight in the range of 305–355 °C. These samples faced a 20% loss of weight in the range of 372–410 °C. They also faced a 50% loss of weight in the range of 423–463 °C, and maximum decomposition observed in the samples occurred at 460–495 °C. The comparative results showed that the chain extender, i.e., BDO, and bioactive material, i.e., xanthan gum, play a vital role. The results illustrated that poly (ethylene glycol adipate)-based polyurethane [33] is thermally less stable compared to xanthan gum, TiO_2_-based polyurethanes with BDO as a chain extender. The values of temperature showed that pure polymers have lower degradation temperature, i.e., 460 °C, while, at the same time, the polymers having 1% XG, 5% of TiO_2_ have higher degradation temperature, i.e., 495 °C, showing degradation temperature variation about 35 °C. Results revealed that the percentage mole ratio of xanthan gum and TiO_2_ enhances the degradation temperature of polyurethane more than pure polyurethane, i.e., XTPU-1. The chain extender, i.e., BDO, and xanthan gum increase the decomposition temperature and thermal stability of XTPU, as shown in Table 2.

#### 4.3.2. Differential Scanning Calorimetry (DSC) Study

**Figure 7:**
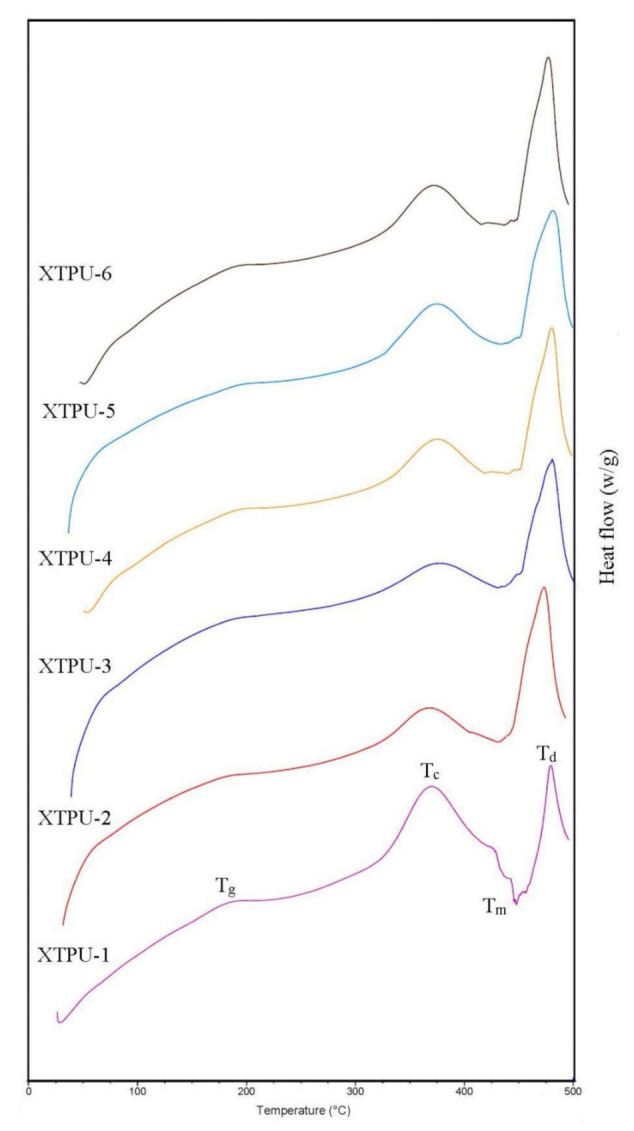
DSC curves of XTPU-1, 2, 3, 4, 5, and 6 at 500 °C.

The effect of chain extenders length, XG, and TiO_2_-based polyurethanes samples were also studied by DSC measurements. The XTPU thermograms are shown in Figure 7. In the XTPU 1 to 6 samples, it is clear by DSC analysis that thermal changes took place when temperature was increased from 0 to 500 °C. It is clear from the data of Table 3 and from DSC thermograms that polymer degradation started in the range of 140–225 °C, which is also in accordance with TGA thermograms. The polymers samples showed glass transition temperature (Tg) in between 178–193 °C, crystallization temperature in the range 368–373 °C, melting temperature (Tm) in the range of 430–450 °C, and decomposition/degradation temperature in the range of 460–495 °C [34]. Here, it has been observed that the melting transition was found to be high near the decomposition temperature. This causes an arrangment of the chains and facilitates a greater interaction between chains, increasing the miscibility between the rigid and soft segment, as seen in TM and Tc, for all materials. The glass transition temperature observed at these values is relative to the rigid segment. The value of the Tg of the polyurethane sometimes also depends on the type of nanofiller (TiO_2_) employed in the synthesis. This value is indicative of the soft and rigid segment mixing degee. The higher value of Tg usually represents the higher miscibilty or campatibity degree of rigid-flexible segments [35]. The results revealed that presence of a nanofiller, i.e., TiO_2_, and its bonding between xanthan gum and diisocyanate, i.e., IPDI, increases the stability and crystalline behavior of the synthesized polymer. So, the crystillinity may be observed because of nanofiller. The most probable reason for this behavior in the nanocomposite is reduction in mobility of chains of urethanes, which reduces the process of degradation, as reported in literature [36]. This can also be related to the formation of longer crosslinked microdomains of rigid segments or the structure with a greater degree of organization. This trend agrees with the results obtained in the literature.

### 4.4. X-Ray Diffraction Study

The crystalline behavior of XTPU samples was calculated by using the crystalline peak intensity of respective samples. The Debye-Scherer (powder) method, applying Bragg’s relation, was used to estimate the d-spacing of various XTPUs [37].

**Figure 8:**
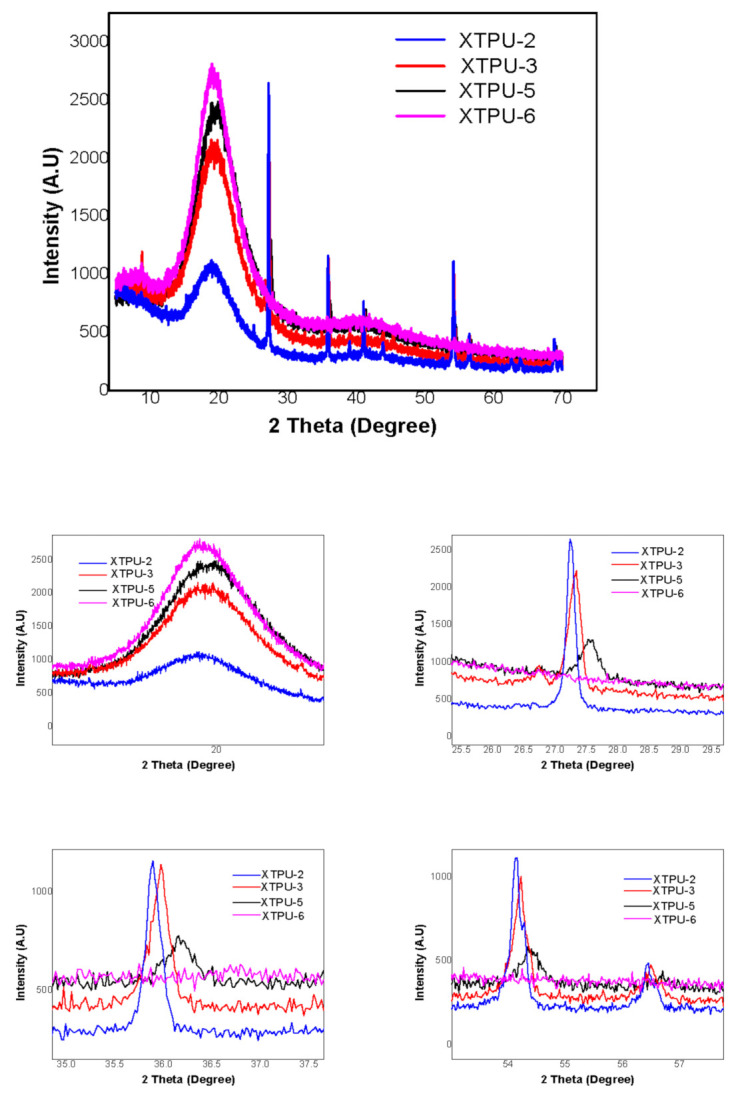
X-ray diffractograms of XTPU-2, XTPU-3, XTPU-5, and XTPU-6 with varying titanium dioxide % age.

In XTPU samples, relative contents, structure regularity, and thermodynamic incompatibility affect the phase separation of soft, as well as hard, segment. A well oriented crystallinity was perceived at 2θ = 20°, as reported in literature [38]. The phase separation of the soft and hard segment in XTPUs is attributed to crystallinity of sample, and it was revealed by X-Ray diffraction studies. So, crystalline behavior improved as % age of TiO_2_ in the final XTPU increased. Finally, in the current study, we can attribute the crystallinity to the soft segment, and increasing percentage of TiO_2_ did not show any appreciable change in polymer structure. The chemical cross-linking of xanthan gum restricts the soft segment melting. So, it can be determined that only XTPU-6, having 1% xanthan gum and 5% TiO_2_, showed higher crystallinity, which has been associated with phase separation at 2θ = 20°, as shown in Figure 8.

### 4.5. Atomic Force Microscopy (AFM)

**Figure 9:**
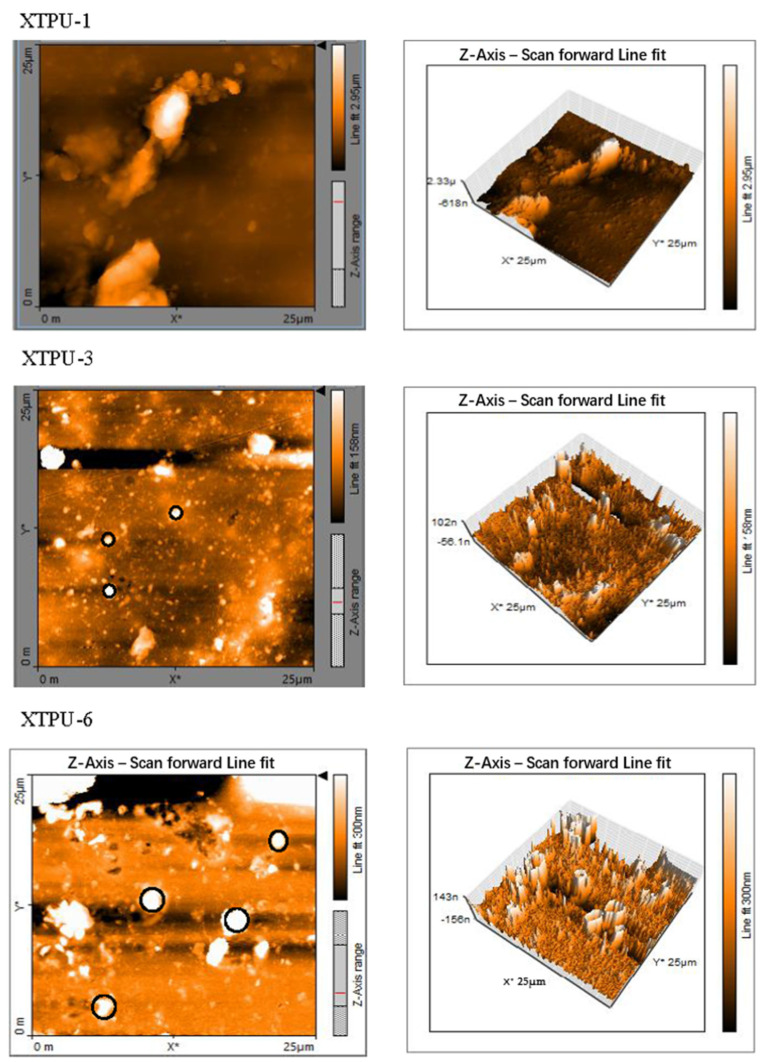
Three-dimensional AFM images of XTPU-1, XTPU-3, and XTPU-6 with 0%, 2%, and 5% titanium dioxide, respectively.

The change in surface morphology of XTPU samples was mainly investigated by AFM. It provides important information relating surface characterization. In the current study, the final morphology of XTPUs films is visualized in Figure 9. In these phase images, the darker regions correspond to soft segments, while lighter domain or crystalline regions correspond to hard segments [2]. The XTPUs can be visualized for the microphase structures as a roughness gradient of 2.5 µm, 158 nm, and 300 nm for XTPU-1, XTPU-3, and XTPU-6 respectively. The complete and better dispersion of nanofiller, also with biomaterial, was also observed within samples. These results showed (the circled region) homogeneous structure of polymer and pattern of distribution of xanthan gum with TiO_2_ in the polyurethane polymer. AFM images of XTPUs have shown that hard segments are completely dispersed in the soft segment matrix. Moreover, it was inferred that, by increasing the content of TiO_2_ from 0% to 5% in 6 polymer samples from XTPU-1 to XTPU-6, the order in pattern crystalline character and stability increases and cracking decreases in the polymer, according to reported literature [25]. Morover, in regard to polyurethane composition, it was also reported that hydrogen bonding was present in urethane groups, which may be the cause of phase segregation. The higher molecular weight of macrodiol and xanthan gum provided ordered and compact arrangments of soft and hard phases. However, various factors can be reponsible for phase segregation in PU, such as the diminsions of soft and hard segments, the polarity of groups, the chemical nature, and molecular weight [37,39].

### 4.6. Evaluation of Water Absorption

The water absorption test and hydrophobicity/hydrophilicity of polyurethane samples are used for evaluation of biodegradability of synthesized polymer. The degree of swelling of polymer components and their affinity to water is one of the indices of decomposition rate of polymeric materials under the influence of environmental factors. Due to presence of soft segment HTPB and XG into the back bone, the prepared polymers are hydrolytic resistant and can be utilized to perform indoor and outdoor environments. So, water absorption capability can be used to determine the hydrolytic degradation of them. The investigation of the hydrolytic stability of XTPUs has shown an increase of the water absorption. Such an increase may be due to the presence of free polar fragments (OH groups), which determine the hydrophilic properties of the polymer. Probably, a part of XG hydrophilic hydroxyls involved in the formation of intermolecular bonds with the polar groups of XTPUs remains unengaged [40]. Water absorption as a function of time and type of samples is summarized in Table 4. Water absorption was calculated by the following formula:

(2) $$\mathrm{Water}\;\mathrm{absorption}\;(\%\;)=\frac{m_{w}-{\;m}_{d}}{m_{d}}\times 100$$

where m_d_ and m_w_ are the masses of dry and wet XTPU samples, accordingly [38]. There was a remarkable water absorption indicated with passage of time in these prepared samples of polyurethanes. The results noticeably indicated that the polymer samples are hydrophilic in nature [41].

## 5. Conclusions

Xanthan gum/Titanium dioxide-based polyurethane elastomers with concordant crystallinity and hydrophilicity were synthezied. The reactants were HTPB, IPDI, and Titanium dioxide that produced prepolymer, extended with BDO. Xanthan gum was used as a bioactive material. Molecular characterization of the prepared elastomers was carried out by FT-IR spectroscopy, and the appearance of NH peaks with disappearance of NCO and OH peaks indicates that the proposed structure of polyurethane has been accomplished. The AFM technique revealed that the degree of micro-phase separation increases with augmenting % age of TiO_2_, which was further confirmed by XRD results. It was revealed by XRD that the crystalline behavior of the synthesized samples due to amorphous region of macrodiol. The % age of TiO_2_ influence on TGA and DSC values gave the information about the thermal stability and thermal changes in the polyurethane samples. The higher value of DSC is due to segments’ mobility in the hard blocks’ microregions (hard domain) and their destruction. Such changes may be caused by three-dimensional XG molecules, which form steric hindrence during polymer formation. Antimicrobial activity determined through the Disc Diffusion Method and the results indicated that the synthesized polyurethane had antimicrobial activity against the *Macrococcus*, *E. coli*, and *Aspergillus flavus*.

## Figures and Tables

**Table 1:** General formulation of polyurethanes.

Sample Code	IPDI (mole)	HTPB (mole)	BDO (mole)	TiO_2_ %	Xanthan Gum %
XTPU-1	2	0.8	1.2	0	0
XTPU-2	2	0.8	1.2	1	1
XTPU-3	2	0.8	1.2	2	1
XTPU-4	2	0.8	1.2	3	1
XTPU-5	2	0.8	1.2	4	1
XTPU-6	2	0.8	1.2	5	1

**Table 2:** Thermal stability data of the XTPU samples based on TGA.

Sample Code	IPDI (mole)	HTPB (mole)	BDO (mole)	TiO_2_ %	X.G %	T_0_ (°C)	T_20_ (°C)	T_50_ (°C)	T_80_ (°C)	T _max_ (°C)
XTPU-1	2	0.8	1.2	0	0	140	372	423	448	460
XTPU-2	2	0.8	1.2	1	1	150	380	430	470	479
XTPU-3	2	0.8	1.2	2	1	162	385	435	475	485
XTPU-4	2	0.8	1.2	3	1	190	372	455	479	487
XTPU-5	2	0.8	1.2	4	1	210	390	457	482	489
XTPU-6	2	0.8	1.2	5	1	225	405	467	485	495

Temperature at which 0%, 20%, 50%, and 80% weight losses obtained from TGA. Maximum decomposition temperature obtained from TGA.

**Table 3:** Thermal stability data of the XTPU samples based on DSC.

Sample Code	IPDI (mole)	HTPB (mole)	BDO (mole)	TiO_2_ %	X.G %	T_g_ (°C)	T_c_ (°C)	T_m_ (°C)	T_d_ (°C)
XTPU-1	2	0.8	1.2	0	0	178	368	430	460
XTPU-2	2	0.8	1.2	1	1	180	365	435	479
XTPU-3	2	0.8	1.2	2	1	183	370	435	485
XTPU-4	2	0.8	1.2	3	1	185	370	440	487
XTPU-5	2	0.8	1.2	4	1	191	371	447	489
XTPU-6	2	0.8	1.2	5	1	193	373	450	495

T_g_ is Glass transition temperature; T_c_ is Crystallization temperature; T_m_ is melting temperature; T_d_ is degradation/decomposition temperature.

**Table 4:** Percentage of water absorption.

Sr. No	Samples Code	Temperature (°C)	% Age of Water Absorption				
			Day 1	Day 2	Day 3	Day 4	Day 5
01	XTPU-1	37	0.94	0.95	0.98	1.10	1.21
02	XTPU-2	37	0.95	0.96	0.99	1.10	1.23
03	XTPU-3	37	0.95	0.98	1.10	1.21	1.31
04	XTPU-4	37	1.10	1.20	1.3	1.5	1.61
05	XTPU-5	37	1.80	1.85	1.90	1.95	1.99
06	XTPU-6	37	1.96	1.98	1.99	2.0	2.1

## Data Availability

The data presented in this study are available on request from the corresponding author.
